# Health impact modelling of different travel patterns on physical activity, air pollution and road injuries for São Paulo, Brazil

**DOI:** 10.1016/j.envint.2017.07.009

**Published:** 2017-11

**Authors:** Thiago Hérick de Sá, Marko Tainio, Anna Goodman, Phil Edwards, Andy Haines, Nelson Gouveia, Carlos Monteiro, James Woodcock

**Affiliations:** a Centre for Epidemiological Research in Nutrition and Health, University of São Paulo, São Paulo, SP, Brazil; b UKCRC Centre for Diet and Activity Research, MRC Epidemiology Unit, University of Cambridge School of Clinical Medicine, Institute of Metabolic Science, Cambridge, UK; c London School of Hygiene and Tropical Medicine, London, UK; d Department of Preventive Medicine, School of Medicine, University of São Paulo, São Paulo, Brazil

## Abstract

**Background:**

São Paulo city, Brazil, faces challenges caused by rapid urbanization. We illustrate how future travel patterns could lead to different health consequences in the city.

**Methods:**

We evaluated the health impacts of different travel pattern scenarios for the São Paulo adult population by comparing the travel patterns of São Paulo in 2012 with counterfactual scenarios in which the city adopted travel patterns of i) those living in the city's expanded centre; ii) London (2012); iii) a highly motorized São Paulo (SP California); and iv) a visionary São Paulo (SP 2040), with high levels of walking and cycling and low levels of car and motorcycle use. For each scenario we estimated changes in exposure to air pollution, road injury risk, and physical activity. Health outcomes were estimated using disability adjusted life years (DALYs) and premature deaths averted. Sensitivity analyses were performed to identify the main sources of uncertainty.

**Results:**

We found considerable health gains in the SP 2040 scenario (total 63.6 k DALYs avoided), with 4.7% of premature deaths from ischemic heart disease avoided from increases in physical activity alone. Conversely, we found substantial health losses in the scenario favouring private transport (SP California, total increase of 54.9 k DALYs), with an increase in road traffic deaths and injuries among pedestrians and motorized vehicles. Parameters related to air pollution had the largest impact on uncertainty.

**Conclusions:**

Shifting travel patterns towards more sustainable transport can provide major health benefits in São Paulo. Reducing the uncertainties in the findings should be a priority for empirical and modelling research on the health impacts of such shifts.

## Highlights

• There is scarce evidence of transportation impacts on health in developing cities
• Comparative risk assessment used to assess impacts on three major risk factors
• We found large net health gains in sustainable transport scenario for Sao Paulo, Brazil
• We also found large net health losses in a car dependent scenario for Sao Paulo
• Physical activity provided the largest health impacts, similar to previous studies

## 1 Background

Population growth and urbanization are projected to add 2.5 billion people to the world's urban population by 2050, with over 90% of the increase concentrated in low-and-middle income countries (United Nations. Department of Economic and Social Affairs. Population Division, 2014). In Latin America and the Caribbean – a region with an already high level of urbanization (80%) – increases in urban population have been followed by a steep rise in motorization rates, particularly in large countries such as Brazil and Mexico (UN-Habitat, 2012). This poses a challenge for the development of sustainable, healthy, safe and equitable transportation systems, centred on walking, cycling and public transport (Whitmee et al., 2015). Adding to the local benefits of the development of sustainable transportation systems (Maizlish et al., 2013, Rojas-Rueda et al., 2011, Woodcock et al., 2009), reducing car and motorcycle dependency has also been suggested as an indispensable strategy to improve planetary health and slow the rate of climate change (Whitmee et al., 2015).

Megacities in the Latin America and the Caribbean region have undergone a chaotic and accelerated process of urbanization in the last century (Gomez et al., 2015, UN-Habitat, 2012). In São Paulo, the largest Brazilian municipality, rapid urbanization has produced a divided city (de Vasconcellos, 2005), with around 10% of the population living in a central wealthier area (hereafter, ‘expanded centre’ or EC) surrounded by the remaining 90% living in a poorer peripheral ‘belt’ (PB). Relative to PB, EC enjoys several features that would seem to favour more sustainable travel patterns. These features include better infrastructure for walking and cycling, better availability of public transport, lower violence rates and more mixed land use. Despite this, travel among residents in EC has historically been dominated by car use (de Vasconcellos, 2005, Rolnik and Klintowitz, 2011) while that among residents of PB is more reliant on public transport. This prominence of public transport in PB is, however, less a consequence of the quality of public transport and more a reflection of resource constraints and lack of alternatives.

São Paulo's future travel pattern, affected by contradictory trends and internal patterns, is hard to predict because policies and influences at different governmental levels are pushing it in different directions – particularly since 2010. At the city and regional level, policies supportive of a more sustainable transport system include the city's Master Plan revision, the development of a long-term plan “*SP 2040: the city we want*” and the discussions around the Municipal Urban Mobility Plan, all of which favour of a less car dependent and less segregated city (Prefeitura de São Paulo, 2012, Prefeitura de São Paulo, 2014a, Prefeitura de São Paulo, 2014b). Related initiatives include the expansion of the cycling and walking infrastructure, extending free bus rides from elderly to school children, and the introduction of strategies for traffic speed reduction. On the other hand, the city travel pattern might also be affected by factors operating at the state and national level. Examples of the former are the recurrent delays in the expansion of metro and trains beyond middle-class areas (Kezič and Durango-Cohen, 2012); and of the latter, desirable increases in purchasing power (particularly among the poor) (de Vasconcellos, 2005, Rolnik and Klintowitz, 2011) combined with tax incentives for vehicle acquisition, which have reinforced car dependency in Brazilian cities, including São Paulo (Amore et al., 2015, Presidência da República, 2010).

The resulting travel patterns from all these policies have large consequences for the health of the citizens. In past years the health benefits and risks of active travel have been extensively studied in international literature (Dhondt et al., 2013, Holm et al., 2012, Macmillan et al., 2014, Maizlish et al., 2013, Rabl and De Nazelle, 2012, Rojas-Rueda et al., 2011, Woodcock et al., 2009, Woodcock et al., 2013, Xia et al., 2015). In these studies the potential health and environmental gains from increases in active travel have been modelled using methods that integrate the benefits of physical activity with the harms from exposure hazards, mainly injury risk and air pollution (Mueller et al., 2015). These studies have consistently found net benefits from active travel when modelling large population changes, although the vast majority of the evidence comes from high-income cities (Mueller et al., 2015), with only one study involving a low or middle income setting (Delhi, India) (Woodcock et al., 2009). Since the magnitude of the estimated health effects of different policies is considerably influenced by baseline levels of physical activity, road traffic injury risk, and air pollution; by the distribution of these risk factors across age and gender; and by the population demographic profile, the net benefit from active travel scenarios in low-and-middle income settings remains unclear.

Therefore, our study aims to create alternative counterfactual scenarios for the city of São Paulo to understand the potential range of the magnitude of health impacts from changes in population travel patterns. To do so, we built two scenario based on travel patterns from observed settings and two scenarios based on hypothetical travel patterns.

## 2 Methods

The study as approved by the Ethics Committee of the School of Public Health, University of São Paulo (process number: 33920).

### 2.1 Description of scenarios

We compared the travel patterns of São Paulo in 2012 (SP 2012/baseline) against the counterfactual scenarios in which the whole city adopted travel patterns:

i) from those living in the centre of the city (SP EC, see Fig. S1), which represents 10% of São Paulo residents. Details from SP observed settings (SP 2012/baseline and SP EC) can be seen in Table 1;

**Table 1:** Characteristics of São Paulo city and São Paulo Central Area.

Variable	São Paulo City	São Paulo Central Area
Population (million)	10.2	1.1
Area (km^2^)	1509	113
Population density (residents/km^2^)	6757	9572
% with college degree	18.9	45.2
Family income (mean per family, R$^a^)	3345	5884
Cars (per 100 household)^b^	68	87
Motorcycle (per 100 household)^b^	9	7
Bicycles (per 100 household)^b^	38	40

Source for São Paulo Area: http://smdu.prefeitura.sp.gov.br/historico_demografico/tabelas.php.

a R$: Brazilian Reais (U$ 100 = R$ 200.9 in July 2012).

b The proportion of households free of cars, motorcycles and bicycles in 2012 are, respectively, 47%, 92% and 73% for São Paulo city and 36%, 94% and 74% for São Paulo central area.
ii) from London in 2012 (SP London 2012), with high levels of public transport use and walking for a high income city (although levels are lower than in São Paulo). In comparison to SP 2012/baseline, London is a more motorized city (78 cars per 100 households in 2013), with similar travel time budget and size; better public transport, road and pedestrian infrastructure; and lower rates of violence and spatial segregation. The travel pattern of London was derived from the London Travel Demand Survey, 2009–2013, a representative population-based survey of London residents using one-day travel diaries;
iii) from a highly motorized São Paulo (SP California), with similar levels of walking, car and public transport use to that observed in California, USA (Götschi et al., 2015), combined with the current levels of cycling and motorcycling of São Paulo in 2012. In comparison to SP 2012/baseline, SP California has higher levels of car and motorcycle use and lower levels of walking and public transport;
iv) from a vision of São Paulo (SP 2040) with much higher levels of walking and cycling and lower levels of car and motorcycle use than today. We quantified this scenario based on several official documents – including the municipality's long term plan “*SP 2040: the city we want*” (Prefeitura de São Paulo, 2012) – as well as social movements' aspirations (Prefeitura de São Paulo, 2012, Prefeitura de São Paulo, 2014b, Soares et al., 2015). SP 2040 would illustrate the travel pattern of a city in which the vast majority of destinations could be reached in no longer than 30 min (Prefeitura de São Paulo, 2012); walking is the main travel mode for both genders and all age groups (Prefeitura de São Paulo, 2012, Prefeitura de São Paulo, 2014b); cycling accounts for 7% of total travel time for both sexes (Soares et al., 2015) (Prefeitura de São Paulo, 2014b), only declining in older age to 4.5% after age 70 and to 1.5% after age 80 (equivalent to reductions observed in the Netherlands (Götschi et al., 2015)); and 70% of travel time from motorized trips comes from public transport. We used the travel pattern from trips no longer than 30 min in SP 2012/baseline as a baseline distribution to build SP 2040 travel pattern. In order to facilitate comparison across scenarios, we used SP 2012/baseline population also for SP 2040.

We assumed population size, age structure and gender split would remain constant in all scenarios in order to isolate the effect of the proposed policy scenarios from changes of demographics.

### 2.2 Integrated Transport and Health Impact Model (ITHIM)

The changes in population health due to changes in active travel time, exposures of air pollution and road traffic injury risk were estimated with the Integrated Transport and Health Impact Model (ITHIM) model. ITHIM has been used to estimate the health effects of transport scenarios and policies at the urban and national level. Different versions of ITHIM have been applied for this purpose in large cities (Maizlish et al., 2013, Woodcock et al., 2009), including one from a low-and-middle income region (Woodcock et al., 2009). Our analysis was conducted on ITHIM's most recent version, able to model the health impact of active travel through three pathways (physical activity, air pollution and traffic injuries) as well as the variability and uncertainty of parameters with Monte Carlo simulation (with 10,000 iterations). The model was implemented in Analytica version 4.6 (Lumina) and is available from the corresponding author upon request. Data analyses were performed in Excel 2010 (Microsoft) and Stata 12 (STATA Corporation). A summary of data sources used to build São Paulo ITHIM version is available at Table 2.

**Table 2:** Characteristics of sources used to build the scenarios for São Paulo.

Characteristics	Household Travel Survey	Health Survey	Road traffic injury	Air pollution
Name	Pesquisa de Mobilidade 2012	Inquérito Domiciliar de Saúde (ISA-Capital)	Sistema de Acidentes de Trânsito - Companhia de Engenharia de Tráfego (SAT-CET)	Qualidade do Ar no Estado de São Paulo (2012)/Emissões Veiculares no Estado de São Paulo (2013)
Frequency	Every five years since 1997	2003 and 2008	Annually	Annually
Year(s) analysed	2012	2008	2009–2013	2012 (PM2.5 concentration) and 2013 (emissions by vehicle type)
Survey size (subjects)	24,534	2691	Around 33,000 victims (1250 fatal) per year	…
Geographic coverage	São Paulo Metropolitan Area	São Paulo city	São Paulo city	São Paulo State
Survey method	Face to face interviews + 1-day travel diary	Face to face interviews. IPAQ^a^ used for physical activity data	Data collection on Police records and at the Institute of Forensic Medicine.	Air quality monitoring network (15 stations for particulate matter in São Paulo city)/Emission inventory based on vehicle fleet, fuel source and emission factors
Age range analysed	18 +	18 +	Any	…

a IPAQ: International Physical Activity Questionnaire.

### 2.3 Travel patterns

We used data collected for the 2012 São Paulo Metropolitan Area Household Travel Survey (SP-HTS) to produce estimates representative São Paulo city itself and to develop the scenarios. More details on the SP-HTS can be found in the Supplementary file.

Travel patterns were modelled as changes based on travel time distribution by mode for each scenario applied to the constant total travel time of São Paulo in 2012. Travel behaviour was modelled as population wide distributions of travel times spent in different modes, stratified by sex and age groups for São Paulo 2012 baseline and each scenario. We assumed the total travel time budget to remain constant in all scenarios.

The health impact from physical activity changes due to changes in travel patterns was modelled in ITHIM using disease specific relative risks (RR) applied to both morbidity and mortality. The same relative risks were assumed for estimating deaths, years of life lost (YLL), and years of healthy life lost due to disability (YLD) in each scenario. We assumed the Sao Paulo background for deaths taken from the Universal Health System (TABNET-SUS)^a^ and the Brazilian background for YLL and YLD taken from the Global Burden of Disease Study (IHME, 2016),^b^ scaled down for the age and sex specific demographic profile equivalent to the population of Sao Paulo The dose-responses used were modelled as normal distributions with RR = 0.84 (standard deviation (sd) = 0.03) for stroke, ischemic heart disease, and other cardiovascular and circulatory diseases (Hamer and Chida, 2008); RR = 0.83 (0.04) for type II diabetes (Jeon et al., 2007); RR = 0.96 (0.02) for depression (Paffenbarger et al., 1994); RR = 0.72 (0.07) for dementia and Alzheimer's disease (Hamer and Chida, 2009); RR = 0.94 (0.01) for breast cancer (Monninkhof et al., 2007); and RR = 0.80 (0.08) for colon cancer in men and RR = 0.86 (0.06) for colon cancer in women (Harriss et al., 2009). See (Götschi et al., 2015) for corresponding exposure marginal METh/week (see below for explanation) and for further details.

There is uncertainty around the exact relationship between physical activity and health outcomes although some studies suggest it as curvilinear, flattening at higher levels of physical activity (Wen et al., 2011, Woodcock et al., 2011). Given the absence of evidence on the exact shape of these disease specific dose-response curves, we assumed a log-linear relationship with power transformations ranging from 0.25 (most curvilinear) to 1 (linear). Sensitivity of the model to shape uncertainty was tested in sensitivity analysis.

### 2.4 Non-travel related physical activity

Age (18 +) and gender specific information on non-travel related physical activity – assumed constant across scenarios – was estimated in Metabolic Equivalent Tasks (METs) using the International Physical Activity Questionnaire (IPAQ) data from the São Paulo City Health Survey (ISA-Capital), which is representative of São Paulo city. METs were converted into marginal METs by subtracting 1 MET. To avoid overestimation we cleaned and analysed IPAQ data following the recommendations of the IPAQ scoring protocol (http://www.ipaq.ki.se; see also (Hagströmer et al., 2008, Ogilvie et al., 2008)). We used the leisure domain to represent non-travel physical activity since most of the cohort studies from which relative risks were taken do not include occupational or domestic activity.

### 2.5 Road traffic injury

We analysed road traffic injury data from 2009 to 2013 obtained by the SAT-CET in the Police records and the Institute of Forensic Medicine. SAT-CET is the official dataset of road traffic injuries in São Paulo. A ‘fatal’ injury is defined as a death resulting from the collision. Most the fatalities (93%) occur within 30 days after the collision. A ‘non-fatal’ injury is defined as any injury for which the victim needed medical assistance or was removed to a health service facility and had the injury reported in the police records. According to the definition used by the SAT-CET, we assumed all ‘non-fatal’ injuries to be serious injuries. In the calculation we used the mean annual numbers between 2009 and 2013.

For each victim we had information about age, gender, mode of transport, location and year. Injuries resulted either from a collision between a striking vehicle and a victim vehicle occupant (or pedestrian) or from a collision in the absence of another vehicle. For collisions with multiple victims in different vehicles, we assumed the largest one to be the striking vehicle for victims in any other vehicle or for pedestrians. For any victims in the largest vehicle we assumed the striking vehicle to be the second largest vehicle. Vehicle size order was defined as follows from smallest to largest: pedestrian, bicycle, motorcycle, car/taxi, pickup/light goods vehicle, bus, and heavy goods vehicle.

The number of injuries was modelled for each scenario based on changes to time travelled by both striking vehicle and victim modes. Gender differences in road traffic injury risks were maintained in the subsequent scenarios, with the impacts of these risks upon numbers of injuries changing in line with changes in travel patterns. Changes in traffic injuries from baseline indexed by age, gender and victim mode were modelled for São Paulo in terms of deaths, YLL, YLD, and disability adjusted life years (DALYs = YLL + YLD). We used empirical estimates from (Elvik, 2009) to consider a potential safety in numbers effect of increases in time travelled by both striking vehicle and victim modes.

### 2.6 Air pollution

We obtained information on concentrations of particulate matter smaller than 2.5 μm (PM2.5) and source apportionment (for primary PM) available for São Paulo (Companhia Ambiental do Estado de São Paulo, 2013b, Santos Junior, 2015). We also used published data on the relative contribution in PM2.5 concentrations by vehicle type (Companhia Ambiental do Estado de São Paulo, 2013a) – 69% for trucks, 23% for buses, 5% for motorbikes and 3% for cars. The relative contribution of transport to total PM2.5 is approximately 60% (Santos Junior, 2015). We then multiplied the time travelled by mode in each scenario to the relative contribution of each vehicle type to obtain the counterfactual PM2.5 concentration. We assumed that the relative contribution of each vehicle type would remain constant across scenarios. For the São Paulo subway and train network, we averaged the PM2.5 values recorded across different stations (Fujii, 2006) and applied this average to the whole system. We did not model the effect of changes in traffic congestion, speed and micro-environment on air pollution. The health impacts of changes in annual PM2.5 exposure were modelled using the integrated exposure-response function proposed by (Burnett et al., 2014) and presented by specific disease in adults: ischemic heart disease, cerebrovascular disease (stroke), chronic obstructive pulmonary disease, and lung cancer. We also estimated the carbon dioxide (CO_2_) equivalent greenhouse gases vehicle emissions (CO_2eq_) for São Paulo city. More details can be found in the Supplementary file.

### 2.7 Sensitivity analysis

We performed several sensitivity analyses to assess the robustness of our findings as well as the influence of different assumptions about key parameters of the model in each scenario. These parameters included the shape of dose-response curves for physical activity, as described above; the disease-specific RR for incidence and mortality; the levels of non-travel physical activity; the MET values assigned for walking and cycling; the exposure response function for PM2.5; the fractions of PM2.5 emissions by mode; the safety-in-numbers power values and the proportion of lifelong or underreported injuries. For sensitivity analysis we also modelled the impact of physical activity directly on all-cause mortality instead of on individual diseases. For this, we used a dose-response function taken from a large cohort study (Wen et al., 2011), two different RRs (for total physical activity and for walking alone) taken from a systematic review (Woodcock et al., 2011), and the RR for walking alone recommended for the WHO Health and Economic Assessment Tool (HEAT) (Kahlmeier et al., 2011). We also modelled the impact of air pollution on all-cause mortality, using the exposure response function for all-cause mortality recommended by (Héroux et al., 2015), with RR = 1.062 (1.040–1.083) per 10 μg/m^3^ PM2.5 exposure. The results of all sensitivity analyses performed are presented in the Supplementary file.

## 3 Results

### 3.1 Travel patterns

**Fig. 1:**
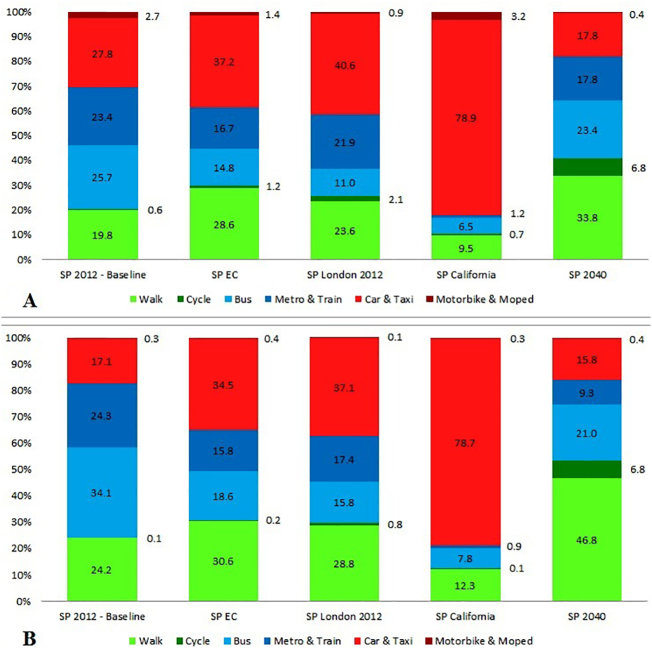
Distribution (%) of daily average travel time by mode for each scenario (1A: Men; 1B: Women). For absolute values, see Table S1.

The travel pattern in EC is close to that of London (Fig. 1). In both areas, car use accounts for over a third of total travel time and the relative contribution of public transport to total travel time is almost 20 percentage points lower than in the entire city SP 2012/baseline. This reflects the fact that in EC and London, income levels and car ownership are higher than the entire Sao Paulo while public transport offer is still high in comparison to other settings such as California (Fig. 1). Cycling levels in London are much higher in relative terms than those from any existing São Paulo setting (EC or the entire city), and the gender gap in cycling is also narrower in London, although still substantial (male:female ratio of cycling 2.8 in London versus 9.1 in São Paulo). In SP California, almost 80% of all trips are made by car whereas less than 10% are made by public transport, both for men and women. In SP 2040, gender cycling inequity is eliminated, with an average of 4 min per day for both men and women. The absolute values for total travel time used to derive the travel patterns are presented in Table S1.

Age distributions of walking and cycling are important for health modelling as changes in physical activity in different age groups might lead to substantially different health impacts. In all scenarios, levels of walking decline with increasing age (Fig. S2). Walking levels are low in SP California for all age groups. SP 2040 is the scenario with the highest levels of walking among the 60 + year old, at around 17 min per day, lower than the current levels in Switzerland (around 33 min per day) and higher than in Netherlands (around 13 min per day) for this age group (Götschi et al., 2015). Cycling levels also decline with increasing age from age group 30–44, being virtually absent in the elderly in all scenarios, except in SP 2040 (Fig. S3). SP 2040 is the scenario with the highest levels of cycling for any age group, with mean time per person per day similar to the current level in Switzerland (Götschi et al., 2015).

### 3.2 Health impact modelling

**Fig. 2:**
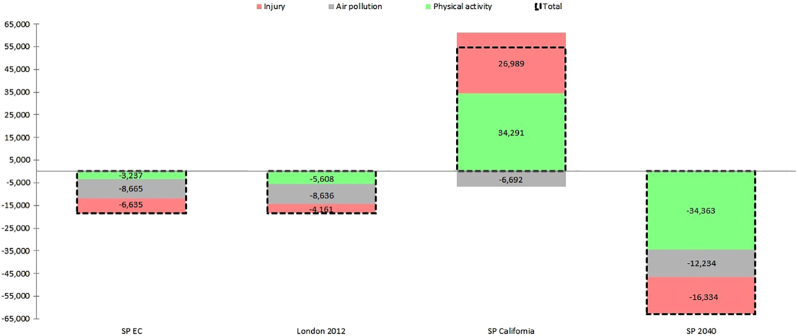
Changes in DALYs for each scenario, broken down into the proportions attributable to changes from air quality, physical activity and road injuries. Detailed results in Table S2.

The health impacts for the São Paulo population of adopting different travel patterns are presented in Fig. 2 as DALY changes from baseline (since DALYs represent loss in health the negative values for DALYs represent a health gain; full results in Table S2). We found net health harms only in the SP California scenarios, mainly driven by reductions in physical activity and increases in road injuries (Fig. 1). SP EC, SP London 2012 and SP 2040 showed positive net benefits with consistent health gains overall as a result of increases in physical activity and reductions in air pollution and road injuries, both for men and women (Fig. 1 and Table S2).

### 3.3 Physical activity

In 2012, around 768,000 DALYs and 27,000 lives were prematurely lost in São Paulo city due to diseases associated with physical inactivity (cardiovascular diseases, breast and colon cancer, type-2 diabetes, dementia, and depression).

An important shift in the disease burden was observed for cardiovascular diseases – particularly ischemic heart disease – and type II diabetes both for men and women (Table 3). For instance, by assuming the travel pattern of SP California, São Paulo would have about 800 extra deaths from reductions in physical activity, of which nearly 700 would be from cardiovascular diseases. The changes in the disease burden favoured women in all scenarios, except for SP EC scenario, mostly due to lower baseline non-travel physical activity levels. In SP 2040, the health gains from physical activity among women are nearly twice that of men both for DALYs and deaths given the large increase in cycling among women in that scenario. The relative contribution of walking and cycling on total marginal METh of physical activity is presented in Table S3.

**Table 3:** Health impact (deaths and DALYS) by disease and gender from changes in physical activity for each scenario (Median (% change from baseline)).

	SP EC	SP London 2012	SP California	SP 2040
Men (deaths)				
Stroke	-4 (− 0.1%)	− 28 (− 1%)	118 (4.3%)	− 94 (− 3.4%)
Ischemic heart disease	− 11 (− 0.2%)	− 39 (− 0.8%)	222 (4.6%)	− 165 (− 3.4%)
Other cardiovascular and circulatory diseases	− 5 (− 0.2%)	− 24 (− 0.8%)	135 (4.6%)	− 98 (− 3.4%)
Type-2 diabetes	− 3 (− 0.3%)	− 11 (− 1%)	47 (4.5%)	− 38 (− 3.6%)
Colon cancer	− 1 (− 0.1%)	− 4 (− 0.5%)	17 (2.3%)	− 14 (− 1.9%)
Dementia and Alzheimer's disease	2 (0.5%)	− 6 (− 1.6%)	8 (2.1%)	− 11 (− 2.9%)
Depression	0 (0%)	0 (0%)	0 (0%)	0 (0%)
Men (DALYs)				
Stroke	− 371 (− 0.4%)	− 498 (− 0.6%)	4189 (5%)	− 2793 (− 3.3%)
Ischemic heart disease	− 602 (− 0.5%)	− 580 (− 0.5%)	6426 (5.1%)	− 4112 (− 3.3%)
Other cardiovascular and circulatory diseases	− 235 (− 0.5%)	− 235 (− 0.5%)	2432 (5.2%)	− 1526 (− 3.2%)
Type-2 diabetes	− 296 (− 0.6%)	− 84 (− 0.2%)	3145 (5.9%)	− 1726 (− 3.3%)
Colon cancer	− 44 (− 0.3%)	− 35 (− 0.3%)	385 (2.7%)	− 260 (− 1.9%)
Dementia and Alzheimer's disease	9 (0.1%)	− 189 (− 1.3%)	353 (2.5%)	− 411 (− 2.9%)
Depression	− 280 (− 0.8%)	84 (0.2%)	1779 (5.1%)	− 721 (− 2.1%)
Women (deaths)				
Stroke	− 5 (− 0.2%)	− 55 (− 1.9%)	66 (2.3%)	− 177 (− 6.2%)
Ischemic heart disease	− 8 (− 0.2%)	− 79 (− 2%)	82 (2.1%)	− 243 (− 6.3%)
Other cardiovascular and circulatory diseases	− 4 (− 0.1%)	− 67 (− 2.1%)	65 (2%)	− 200 (− 6.1%)
Type-2 diabetes	− 3 (− 0.2%)	− 27 (− 2.1%)	29 (2.3%)	− 84 (− 6.6%)
Colon cancer	− 1 (− 0.1%)	− 6 (− 0.7%)	9 (1%)	− 22 (− 2.5%)
Breast cancer	− 2 (− 0.2%)	− 6 (− 0.5%)	25 (1.9%)	− 37 (− 2.9%)
Dementia and Alzheimer's disease	2 (0.2%)	− 19 (− 2.2%)	0 (0%)	− 40 (− 4.6%)
Depression	0 (0%)	0 (0%)	0 (0%)	0 (0%)
Women (DALYs)				
Stroke	− 284 (− 0.4%)	− 967 (− 1.3%)	2856 (3.8%)	− 4728 (− 6.3%)
Ischemic heart disease	− 436 (− 0.4%)	− 1429 (− 1.4%)	3817 (3.7%)	− 6649 (− 6.4%)
Other cardiovascular and circulatory diseases	− 150 (− 0.4%)	− 529 (− 1.3%)	1646 (3.9%)	− 2647 (− 6.3%)
Type-2 diabetes	− 305 (− 0.5%)	− 639 (− 1%)	3006 (4.7%)	− 4201 (− 6.6%)
Colon cancer	− 28 (− 0.2%)	− 60 (− 0.4%)	247 (1.6%)	− 380 (− 2.5%)
Breast cancer	− 55 (− 0.2%)	− 64 (− 0.2%)	713 (2.5%)	− 819 (− 2.9%)
Dementia and Alzheimer's disease	− 18 (− 0.1%)	− 338 (− 2%)	156 (0.9%)	− 861 (− 5%)
Depression	− 135 (− 0.3%)	− 25 (0%)	2503 (4.9%)	− 2287 (− 4.4%)

### 3.4 Road traffic injuries

In the five years, 2009 and 2013, São Paulo had over 150,000 injuries and 5000 deaths from road traffic. For our baseline scenario (SP 2012/baseline), the estimates were 42,896 injured and 1126 deaths, respectively. Gender differences in numbers of injuries and fatalities for cycling and motorcycling were substantial, against men, reflecting a much higher use of these travel modes among men (Fig. 1 and Table S1). By contrast, the observation that the number of deaths among male pedestrians is twice that among females cannot be explained by gender differences in walking time but may be related to differences in where and how they walk as well as differences in other risk-related behaviours, such as higher alcohol consumption among men. Gender differences in road traffic injury risks were reflected in the subsequent scenarios, influenced by changes in travel patterns (Table 4).

**Table 4:** Number of fatal and serious injuries by mode of travel and gender for each scenario (Median (% change from baseline)).

	SP EC	SP London 2012	SP California	SP 2040
Men, fatal				
Pedestrian injury	− 29 (− 8%)	− 14 (− 4%)	25 (7%)	− 101 (− 29%)
Cyclist injury	3 (7%)	55 (143%)	5 (14%)	230 (600%)
Motorcycle and mopeds injury	− 118 (− 36%)	− 145 (− 44%)	143 (44%)	− 293 (− 90%)
Car, van, bus and truck injury	− 2 (− 1%)	20 (11%)	143 (79%)	− 86 (− 48%)
Other road and transport injury	0 (− 14%)	0 (− 2%)	0 (− 3%)	0 (5%)
Women, fatal				
Pedestrian injury	− 14 (− 9%)	− 8 (− 5%)	13 (8%)	− 48 (− 31%)
Cyclist injury	0 (− 2%)	0 (24%)	0 (− 22%)	2 (131%)
Motorcycle and mopeds injury	− 10 (− 34%)	− 13 (− 43%)	8 (25%)	− 27 (− 90%)
Car, van, bus and truck injury	− 1 (− 2%)	6 (14%)	45 (108%)	− 20 (− 49%)
Other road and transport injury	0 (− 8%)	0 (− 3%)	0 (4%)	0 (− 20%)
Men, serious				
Pedestrian injury	− 106 (− 2%)	573 (11%)	1862 (36%)	− 1401 (− 27%)
Cyclist injury	226 (16%)	3383 (232%)	661 (45%)	14,168 (971%)
Motorcycle and mopeds injury	− 4202 (− 28%)	− 6202 (− 41%)	11,028 (72%)	− 12,972 (− 86%)
Car, van, bus and truck injury	369 (5%)	2485 (31%)	12,369 (154%)	− 3074 (− 38%)
Other road and transport injury	0 (− 1%)	3 (10%)	8 (30%)	− 4 (− 15%)
Women, serious				
Pedestrian injury	− 154 (− 3%)	374 (8%)	1579 (34%)	− 1319 (− 28%)
Cyclist injury	14 (17%)	150 (177%)	39 (46%)	555 (654%)
Motorcycle and mopeds injury	− 596 (− 24%)	− 1052 (− 42%)	1381 (55%)	− 2117 (− 85%)
Car, van, bus and truck injury	− 192 (− 3%)	1112 (20%)	6680 (121%)	− 2331 (− 42%)
Other road and transport injury	0 (0%)	1 (10%)	2 (32%)	− 1 (− 15%)

As shown in Table 4, a large increase in car levels also showed increases in pedestrian injuries or deaths, for both sexes (SP California). The scenario also resulted in increases in motorcyclist, car, van, bus and truck fatalities in both sexes. In SP 2040, the substantial increases in deaths from cycling are also noticeable, reflecting much higher bicycle use both for men and women. Despite large uncertainties for injuries in scenarios with substantial cycling increase (e.g. 14,167 (95% Credible Interval: 7833 to 24,920) for men and 555 (95% Credible Interval: 295 to 993) for women in SP 2040), the overall health benefits are positive in all the scenarios for any of the Credible Intervals assumed. SP 2040 is the only scenario with substantial reductions in injuries and death across all other modes than cycling.

### 3.5 Air pollution

Since the relative contribution of buses to PM2.5 concentrations in São Paulo is higher than that of cars or motorcycles, all scenarios showed reductions in background PM2.5 concentrations (see Table 5) as they involved reductions in total public transport by having more cars (SP California), more walking and cycling (SP 2040) or a combination of both (SP EC and SP London 2012) (Table 5).

**Table 5:** Health impact (deaths and DALYS) by gender from changes in air pollution for each scenario (Median (95% Credible Intervals)).

	SP EC	SP London 2012	SP California	SP 2040
Deaths				
Men				
Stroke	− 88 (− 119 to − 61)	− 86 (− 122 to − 55)	− 64 (− 131 to 1)	− 120 (− 156 to − 87)
Ischemic heart disease	− 56 (− 81 to − 29)	− 54 (− 80 to − 29)	− 44 (− 89 to − 2)	− 76 (− 104 to − 47)
Tracheal, bronchus and lung cancer	− 11 (− 17 to − 6)	− 12 (− 18 to − 6)	− 8 (− 19 to 0)	− 18 (− 25 to − 11)
Chronic obstructive pulmonary disease	− 12 (− 16 to − 7)	− 12 (− 17 to − 7)	− 8 (− 17 to 2)	− 16 (− 21 to − 10)
Women				
Stroke	− 80 (− 110 to − 54)	− 82 (− 119 to − 50)	− 43 (− 106 to 19)	− 112 (− 149 to − 75)
Ischemic heart disease	− 33 (− 52 to − 17)	− 34 (− 58 to − 17)	− 17 (− 48 to 11)	− 50 (− 76 to − 27)
Tracheal, bronchus and lung cancer	− 8 (− 13 to − 4)	− 9 (− 14 to − 4)	− 5 (− 13 to 1)	− 13 (− 18 to − 7)
Chronic obstructive pulmonary disease	− 8 (− 11 to − 4)	− 8 (− 12 to − 4)	− 3 (− 11 to 3)	− 11 (− 15 to − 7)
DALYs				
Men				
Stroke	− 2801 (− 3786 to − 1929)	− 2732 (− 3835 to − 1769)	− 2316 (− 4489 to − 230)	− 3907 (− 5067 to − 2849)
Ischemic heart disease	− 1534 (− 2231 to − 768)	− 1476 (− 2160 to − 779)	− 1318 (− 2531 to − 143)	− 2078 (− 2817 to − 1293)
Tracheal, bronchus and lung cancer	− 281 (− 423 to − 145)	− 279 (− 441 to − 143)	− 225 (− 501 to − 16)	− 444 (− 610 to − 279)
Chronic obstructive pulmonary disease	− 365 (− 489 to − 225)	− 360 (− 519 to − 221)	− 293 (− 585 to − 3)	− 505 (− 681 to − 361)
Women				
Stroke	− 2273 (− 3092 to − 1551)	− 2342 (− 3322 to − 1503)	− 1580 (− 3348 to 140)	− 3238 (− 4288 to − 2236)
Ischemic heart disease	− 1031 (− 1535 to − 556)	− 1062 (− 1638 to − 558)	− 717 (− 1627 to 84)	− 1509 (− 2173 to − 888)
Tracheal, bronchus and lung cancer	− 163 (− 247 to − 89)	− 167 (− 270 to − 88)	− 114 (− 272 to 0)	− 263 (− 364 to − 152)
Chronic obstructive pulmonary disease	− 238 (− 325 to − 149)	− 244 (− 350 to − 143)	− 155 (− 353 to 36)	− 337 (− 460 to − 220)
PM2.5 background concentration (μg/m^3^)^a^	16.9 (15.7 to 18.1)	16.7 (15.4 to 17.9)	18 (16.4 to 19.7)	16.1 (14.9 to 17.3)

a Baseline PM2.5 background concentration: 18.5 μg/m^3^ (17.3 to 19.7 μg/m^3^).

Health gains from the reduction in PM2.5 concentrations varied between scenarios due to different levels of exposure across age groups as a result of different travel patterns. For instance, exposure levels in SP London 2012 were higher in the elderly (60 y or more) than in other scenarios. Larger health gains in both deaths and DALYs were observed for cardiovascular diseases (stroke and ischemic heart disease) when compared to other health outcomes for all scenarios. If São Paulo adopts the travel pattern of SP 2040 – with twice the level of active travel and almost half the level of private transport and bus use – a total of 406 deaths would be avoided per year, nearly a third of the number of deaths avoided from increases in physical activity (1224 deaths).

## 4 Discussion

### 4.1 Principal findings

**Fig. 3:**
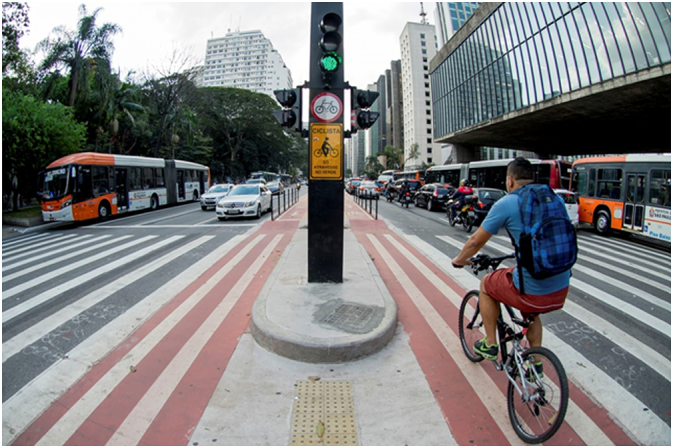
Cycle lane at the Paulista Avenue, São Paulo, built in 2015. Note motorcyclists trafficking between car queues, a common feature in São Paulo, and the dedicated bus lanes next to the sidewalks. Left, the remaining original vegetation that once were in the Paulista Avenue (Trianon Park); Right, the Museum of Arts of São Paulo (MASP). Photo: Marcelo Camargo / Agência Brasil (Fotos Públicas).

In this study we observed substantial population health benefits in São Paulo following a shift to a travel pattern featuring considerably more active travel and less car and motorbike use (SP 2040). These modelled benefits resulted from increasing physical activity and reducing air pollution and road traffic injuries. Extending the travel pattern of the expanded centre to the entire city would also generate health gains, mainly due to reductions in air pollution. Health effects differed by gender across scenarios, with a more favourable trade-off among women. Our study also indicate that adopting the travel pattern of London, UK, would also generate net health benefits for São Paulo, similar to EC and lower than SP 2040. This would come mainly from reductions in air pollution and increases in physical activity, highlighting the importance of the local context when implementing changes. Working to gradually move towards SP 2040 scenario seems a promising way forward to São Paulo (Fig. 3).

### 4.2 Strengths and limitations

At present, ITHIM is the only model developed to assess the health impacts of transport changes that has been applied in any low- and middle-income setting (Mueller et al., 2015), in which availability and quality of primary data tend to be lower than in high-income settings. We used the best available primary data for São Paulo to build the scenarios and to test effects of deviations in key parameters. These analyses showed great uncertainty for parameters still in debate in the literature, such as the functional form of dose-response curves (Wen et al., 2011, Woodcock et al., 2011) or the relative contribution of each mode of transport to PM2.5 concentrations. Model uncertainty is moderate, becoming larger in scenarios assuming the biggest changes in travel pattern (SP California and SP 2040).

ITHIM São Paulo allows estimating the health impact of transport changes through physical activity, air pollution and road traffic injury, pathways most commonly used in this type of assessment (Mueller et al., 2015). However, some important transport-related exposure-outcome associations were not included, such as vehicle speeds, which could affect air pollution due to an association between speed and congestion as well as the number and severity of traffic injuries (Elvik, 2013); traffic noise; and other forms of interpersonal violence related to travel behaviour (e.g., female sexual harassment and road rage episodes). We also did not model changes in vehicle emission factors, particularly cleaner buses for public transport, which if implemented would produce substantial health benefits.

Additional strengths of ITHIM São Paulo include: considering morbidity and not just all-cause mortality; realistic population wide distributions by age and gender of travel times; the inclusion of non-travel physical activity and background disease rates; and mode specific estimations of exposure to air pollution, based on time in traffic/subway and ventilation rates. Recent evidence supports the assumption that increased levels of active travel do not lead to a change in non-travel or leisure-time physical activity, at least in a high income setting (Foley et al., 2015). We did not model the benefits of physical activity on overweight and at younger ages in later life, the longer term effect of reduced air pollution, nor emerging effects such as on cognitive function, low birth weight or, and therefore may have underestimated the net health effects. Particularly for the SP 2040 scenario, results may also be underestimated since we used the 2012 baseline population instead of the projections for 2040 in order to facilitate comparability across scenarios. In 2040, a larger proportion of people in older age groups is expected (Table S6), a group that benefits more from active travel in comparison to younger groups (Mueller et al., 2015) mainly because of their higher risk of chronic diseases (Chodzko-Zajko et al., 2009).

The plausibility of our scenarios can be questioned. In SP 2040, we envisage high cycling levels and the virtual elimination of age and gender inequities in cycling, similar to the Netherlands. Assuming those changes to occur between 2016 and 2040, a large increase in cycling uptake would be needed, which has only been seen in places with strong policies and sustained investments, such as Portland, USA (5-fold increase in mode share in 19 years) (Pucher et al., 2011). Evidence suggests that by itself an increase in the cycling level is not sufficient to reduce age and gender inequities in cycling use (Aldred et al., 2015). For modes other than cycling, the changes modelled in SP 2040 and in other scenarios, although sometimes large, are similar to those observed in several different settings. Moreover, measures to enable active travel and to reduce private transport use may reinforce themselves since access to a private vehicle is the most important factor associated with physical inactivity in transportation in São Paulo residents (Sa et al., 2013).

### 4.3 Comparison against other studies and models

The direction of changes observed for the scenario with increasing active travel and reductions in private transport in São Paulo (physical activity increase, air pollution and road traffic injury reduction) was similar to those found in Delhi. The magnitude of DALYs gained was also similar, with approximately 7.2 k and 11.3 k DALYs gained per million population in Delhi and São Paulo.

The comparison with other studies that included these three health pathways (Mueller et al., 2015) – all in cities from high-income countries – showed discrepancies in the direction of changes only for the road traffic injury pathway, with some studies reporting an increase (Holm et al., 2012, Macmillan et al., 2014, Maizlish et al., 2013, Rabl and De Nazelle, 2012, Woodcock et al., 2009) and some reduction in road traffic injuries (Dhondt et al., 2013, Woodcock et al., 2013, Xia et al., 2015) following changes towards more sustainable transport systems. All studies showing reductions in road traffic injuries, including ours, assumed a non-linearity of risks with increased active travel, e.g., by taking into account a safety-in-numbers effect or variations in the volume of travel by ‘striking vehicle’ modes (Dhondt et al., 2013, Woodcock et al., 2013, Xia et al., 2015). Also of note are the gender differences in road injury risks, which systematically disadvantaged men (smaller gains or larger harms for men), similar to the observed in the Netherlands (Stipdonk and Reurings, 2012) but not in London (Woodcock et al., 2014). Unfortunately, few studies assessing the health impact of transport and urban planning changes address health inequalities (Mueller et al., 2015).

The relative contribution of air pollution to the net health impact of our scenarios was similar to that observed in Delhi (Woodcock et al., 2009) and higher than in most high income settings (Macmillan et al., 2014, Maizlish et al., 2013, Rabl and De Nazelle, 2012, Woodcock et al., 2009, Woodcock et al., 2013, Xia et al., 2015), except for (Dhondt et al., 2013). In all those places, including in our study, physical activity made a major contribution to the health benefits. In São Paulo there are higher levels of non-travel physical activity compared with high income societies probably because they are at different stages in the epidemiological and physical activity transitions (Table S7). This reduces the relative contribution of active travel to total physical activity (Ng and Popkin, 2012). On the other hand, there was little change to the estimates in sensitivity analyses applying much lower baseline levels of non-travel physical activity (Figs. S4 to S7). It is important to note that the annual average PM2.5 concentrations in Delhi were substantially larger than in SP (Woodcock et al., 2009) and therefore the estimates of pollution related health outcomes would be more influenced by the use of the non-linear dose-response function for PM2.5. Finally, our sensitivity analyses for both physical activity and air pollution highlights the findings that modelled effects on all-cause mortality instead of on individual diseases are larger than the combined effects on increased risk of death from individual diseases perhaps because air pollution increases the risk of death from other diseases. Thus studies using that strategy may also find larger results for each pathway as well as for the net health effect, assuming all pathways estimates point to the same direction.

### 4.4 Policy implications

São Paulo should pursue the effective implementation and monitoring of several recent initiatives aiming to enable and facilitate active travel as well as to slow growth of car and motorcycle use (Prefeitura de São Paulo, 2012, Prefeitura de São Paulo, 2014a, Prefeitura de São Paulo, 2014b). Policies to tackle age and gender health disparities in transport, especially in cycling, are of particular relevance to achieve the substantial changes required for a positive health impact. Walking and cycling should be the safest, cheapest, most pleasant and convenient options for most everyday trips, which means shifting priority and investments from roads for motorists towards improvements in favour of active travel and mass transit.

A strong commitment towards sustainable transport would have the potential to halve the CO_2eq_ emissions from road transport (e.g., − 3.6 million tons of CO_2eq_ emissions for SP 2040; see Supplementary file), which is the sector with the single largest contribution, and growing trend of CO_2eq_ emissions in the city (Companhia Ambiental do Estado de São Paulo, 2013a). Such bold reduction is in accordance with the political commitment to a “drastic reduction in the greenhouse gases emissions” (Prefeitura de São Paulo, 2012). At the national level, it could also contribute to the Brazilian State's commitment to reduce the greenhouse gas emissions by 43% by 2030 (based on emissions from 2005) (Brazil, 2015), given the important contribution of São Paulo to national emissions.

Ongoing policies that seek to shift the city in the right direction should be encouraged, with proper evaluation to ensure that the hoped for transport and health benefits are realised. Such policies include the construction of dedicated cycling infrastructure, improvements in sidewalks, larger investments in mass transit, and strategies for traffic-calming and traffic law reinforcement. Other promising initiatives for which net health effects have not been established in low- and middle-income settings include the intended expansion of the cycle hire system for the entire city as well as the vehicle inspection program, discontinued in 2014. Buses could be much cleaner: strategies to reduce the contribution and mitigate the negative impact of public transport on air pollution, such as fleet renewal, adequate fleet maintenance and changes in the energy matrix, could increase the benefits observed. Proper planning for a cleaner public transport is essential so that effective implementation do not lead to unintended consequences, such as reduction of public transport availability and use, as seen in other low- and middle-income cities (Goel and Guttikunda, 2015). Gender differences from road injury against men across scenarios point to the fact that, together with improvements in infrastructure and traffic conditions, it would be indispensable to enact policies to reduce risk of traffic injury, and to improve the working conditions of groups of a higher risk, particularly courier motorcyclists (‘*motoboys*’) (Bacchieri and Barros, 2011).

A better designed and more compact, decentralised and diverse city would support active travel uptake and increase access to other social determinants of health, such as education, work, leisure, green space, services, and health facilities. It would also generate large travel time savings (Sá et al., 2015), in a city with high levels of traffic congestion and daily accumulated travel time. In other words, contrary to traditional transport goals and policies, we should aim for travel time savings not by moving people faster but reducing necessary distances. To create this visionary city, São Paulo and its citizens would have to overcome the long history of segregation and dispossession against the poor population and the privileges traditionally given to the middle class and the elite (Vasconcellos, 2014). Brazil has undergone remarkable reductions in social inequities in the last decade, with the eradication of hunger and improvements in housing, employment and health, particularly among poorer groups. As the country's largest and richest city, São Paulo stakeholders could also lead a national debate and become a role model for how to improve people's wellbeing and quality of life in urban areas through improved transport policies (Amore et al., 2015, Presidência da República, 2010).

### 4.5 Unanswered questions and future research

A number of parameters used came from high-income settings or populations, including the relation between daily and weekly travel behaviour for walking and cycling, the average YLD for lifelong and temporal road injuries (Tainio et al., 2014), the mode-specific ventilation rates, and most of the relative risks used for the physical activity pathway.

Moreover, more studies on low- and middle-income settings would be welcome including to clarifying exposure response relationships for air pollution exposure. Modelling specific policies together with empirical research, in close coordination with policymakers, would also allow a more refined analysis on how to best achieve context-specific changes in population travel patterns. Future research should aim to better understand the relation between travel patterns and other risk factors, such as noise, food behaviour, independent mobility and social isolation as well as other health-relevant issues, such as quality of life, happiness, violence and mental well-being.

## 5 Conclusion

São Paulo has the challenge to overcome the negative consequences of a chaotic and accelerated process of urbanization, and meeting this challenge will require the development of a healthier and more efficient travel pattern. Our results indicate that moving urban trips from car and motorcycle travel to walking, cycling and clean public transport can provide health benefits as long as such changes are substantial and across the whole population. Many of São Paulo's existing plans and initiatives are a promising way forward in that direction and we hope that future research can monitor the extent to which they achieve their aspiration to create a ‘São Paulo we want’.

## Appendix A Supplementary data

Supplementary material Image 1

## Footnotes

a Ministério da Saúde. Departamento de Informática do SUS-DATASUS. Sistema de Informação sobre Mortalidade. Brasília: Informações de Saúde (Tabnet): estatísticas vitais, 2015. Available from http://datasus.saude.gov.br/informacoes-de-saude/tabnet. (Accessed June 5th 2017).

b Institute for Health Metrics and Evaluation (IHME). GBD Compare Data Visualization. Seattle, WA: IHME, University of Washington, 2016. Available from http://vizhub.healthdata.org/gbd-compare. (Accessed June 5th 2017).
