# Supplementary material for: Health impact modelling of different travel patterns on physical activity, air pollution and road injuries for São Paulo, Brazil
Source: Environ Int. 2017 Nov;108:22–31. doi: 10.1016/j.envint.2017.07.009 (PMC5632958; doi:10.1016/j.envint.2017.07.009)
Supplement: Supplementary file 1 — Supplementary material [file mmc1.docx]

**Supplemental Material**

**Health impact modelling of different travel patterns on physical activity, air pollution and road injuries for São Paulo, Brazil.**

**Thiago Hérick de Sá, Marko Tainio, Anna Goodman, Phil Edwards, Andy Haines, Nelson Gouveia, Carlos Augusto Monteiro, James Woodcock**

Table of Contents

[**Table of Contents 1**](#_Toc489124730)

[**Table S3. Relative contribution of active travel (walking and cycling) on total METh from physical activity for each scenario. 5**](#_Toc489124731)

[**Figure S1. Geographic differences between the 6**](#_Toc489124732)

[**Figure S2. Average walking minutes per day by age groups and scenario. 7**](#_Toc489124733)

[**. 8**](#_Toc489124734)

[**Figure S3. Average cycling minutes per day by age groups and scenario. 8**](#_Toc489124735)

[**Table S6. Age and gender distribution of the adult population of São Paulo in 2012 and projections for São Paulo in 2040 (# (% Total)). 13**](#_Toc489124736)

[**Table S7. Average non-travel physical activity by age and gender (marginal METh per week). 14**](#_Toc489124737)

[**Figures S4 to S7. Tornado plots from sensitivity analysis. 15**](#_Toc489124738)

[**Figures S4. Tornado plot for the SP Expanded Centre scenario. 15**](#_Toc489124739)

[**Figures S5. Tornado plot for the London 2012 scenario. 16**](#_Toc489124740)

[**Figures S6. Tornado plot for the SP California scenario. 17**](#_Toc489124741)

[**Figures S7. Tornado plot for the SP 2040 scenario 18**](#_Toc489124742)

[**References in the Supplementary file 19**](#_Toc489124743)

| Table S1. Daily average minutes of travel by mode of transport and gender for each scenario (median values). | | | | | |
| --- | --- | --- | --- | --- | --- |
| Male |  |  |  |  |  |
|  | SP 2012 - Baseline | SP EC | SP London 2012 | SP California | SP 2040 |
| Walk | 18.6 | 21.8 | 18.5 | 7.5 | 19.8 |
| Cycle | 0.6 | 0.9 | 1.6 | 0.6 | 4.0 |
| Bus | 24.1 | 11.3 | 8.6 | 5.2 | 13.7 |
| Car & Taxi | 26.1 | 28.3 | 31.8 | 62.4 | 10.4 |
| Motorbike & Moped | 2.5 | 1.1 | 0.7 | 2.5 | 0.2 |
| Metro & Train | 22.0 | 12.7 | 17.2 | 0.9 | 10.4 |
| Total | 93.9 | 76.1 | 78.5 | 79.1 | 58.4 |
|  |  |  |  |  |  |
| Female |  |  |  |  |  |
|  | SP 2012 - Baseline | SP EC | London 2012 | SP California | SP 2040 |
| Walk | 19.6 | 20.8 | 20.5 | 9.0 | 26.9 |
| Cycle | 0.1 | 0.1 | 0.6 | 0.1 | 3.9 |
| Bus | 27.7 | 12.6 | 11.2 | 5.7 | 12.1 |
| Car & Taxi | 13.8 | 23.5 | 26.4 | 57.8 | 9.1 |
| Motorbike & Moped | 0.2 | 0.3 | 0.1 | 0.2 | 0.2 |
| Metro & Train | 19.7 | 10.8 | 12.4 | 0.6 | 5.3 |
| Total | 81.1 | 68.1 | 71.1 | 73.5 | 57.4 |

*São Paulo Metropolitan Area Household Travel Survey*

To derive the estimates presented in Table S1 in order to develop the scenarios, we used data collected for the São Paulo Metropolitan Area Household Travel Survey (SP-HTS) from 2012, which followed a complex and stratified sampling plan to produce estimates representative for each municipality in the São Paulo Metropolitan area, including São Paulo city itself. To select the households, São Paulo city was divided into 23 traffic zones, which are the smallest area level with statistical representativeness in the survey. We defined the EC as the merged area from five zones, corresponding to 20 central districts out of the 96 city districts. We chose to use zones to build EC and not the municipal official boundaries of the expanded central area because the zones are the primary sampling unit from the SP-HTS and also represent territories (‘districts’) with local structures of governance (see Figure S1 for the geographical differences).

SP-HTS data were collected for every household member using a face-to-face interview on various days of the week to have all weekdays represented in the sample. Trip-level data consisted of one-way trips undertaken on the day before the interview was held, and included trip purpose, origin and destination, time of departure and time of arrival, mode of transport, and the number of changes between transport modes during the trip. Further information about SP-HTS can be obtained elsewhere (Companhia do Metropolitano de São Paulo 2013; Sá et al. 2015).

| Table S2. Changes in DALYs for each scenario, broken down into the proportions attributable to changes from air quality, physical activity and road injuries (Median (95% Credible Intervals) | | | | |
| --- | --- | --- | --- | --- |
|  | SP EC | SP London 2012 | SP California | SP 2040 |
| Men, DALYs |  |  |  |  |
| Physical activity | -1826 (-3568 to -945) | -1548 (-5701 to 1250) | 19014 (13170 to 25567) | -11633 (-21157 to -5792) |
| Air pollution | -4964 (-6810 to -3267) | -4837 (-6872 to -3095) | -4126 (-8020 to -556) | -6899 (-9106 to -4903) |
| Injury | -5658 (-8760 to -3725) | -3852 (-6193 to -2279) | 21065 (11752 to 35345) | -11980 (-18921 to -6638) |
| Women, DALYs |  |  |  |  |
| Physical activity | -1415 (-2601 to -773) | -4091 (-7655 to -1862) | 15241 (10842 to 20541) | -22720 (-37218 to -13917) |
| Air pollution | -3699 (-5142 to -2441) | -3806 (-5505 to -2377) | -2575 (-5500 to 190) | -5331 (-7246 to -3556) |
| Injury | -977 (-1520 to -629) | -313 (-449 to -161) | 5916 (3009 to 10594) | -4412 (-6991 to -2683) |
| Men and Women, DALYS |  |  |  |  |
| Physical activity | -3237 (-6170 to -1720) | -5608 (-13341 to -752) | 34291 (24143 to 45907) | -34363 (-58368 to -19762) |
| Air pollution | -8665 (-11962 to -5732) | -8636 (-12402 to -5481) | -6692 (-13519 to -402) | -12234 (-16344 to -8471) |
| Injury | -6635 (-10264 to -4359) | -4161 (-6636 to -2460) | 26989 (14800 to 45970) | -16334 (-25414 to -10110) |

| Table S3. Relative contribution of active travel (walking and cycling) on total METh from physical activity for each scenario. | | | | |
| --- | --- | --- | --- | --- |
|  | Cycling | Walking | All other | Total |
| SP 2012 - Baseline |  |  |  |  |
| Male | 2.0% | 36.1% | 19.4% | 57.4% |
| Female | 0.2% | 36.4% | 6.0% | 42.6% |
| Total | 2.2% | 72.5% | 25.4% | 100.0% |
| SP EC |  |  |  |  |
| Male | 2.6% | 37.9% | 17.8% | 58.3% |
| Female | 0.2% | 36.0% | 5.5% | 41.7% |
| Total | 2.9% | 73.8% | 23.3% | 100.0% |
| SP London 2012 |  |  |  |  |
| Male | 5.4% | 34.4% | 16.8% | 56.6% |
| Female | 2.0% | 36.3% | 5.2% | 43.4% |
| Total | 7.4% | 70.7% | 21.9% | 100.0% |
| SP California |  |  |  |  |
| Male | 3.2% | 26.6% | 30.4% | 60.2% |
| Female | 0.3% | 30.1% | 9.4% | 39.8% |
| Total | 3.5% | 56.8% | 39.7% | 100.0% |
| SP 2040 |  |  |  |  |
| Male | 10.7% | 27.5% | 12.2% | 50.4% |
| Female | 10.7% | 35.1% | 3.8% | 49.6% |
| Total | 21.5% | 62.6% | 15.9% | 100.0% |


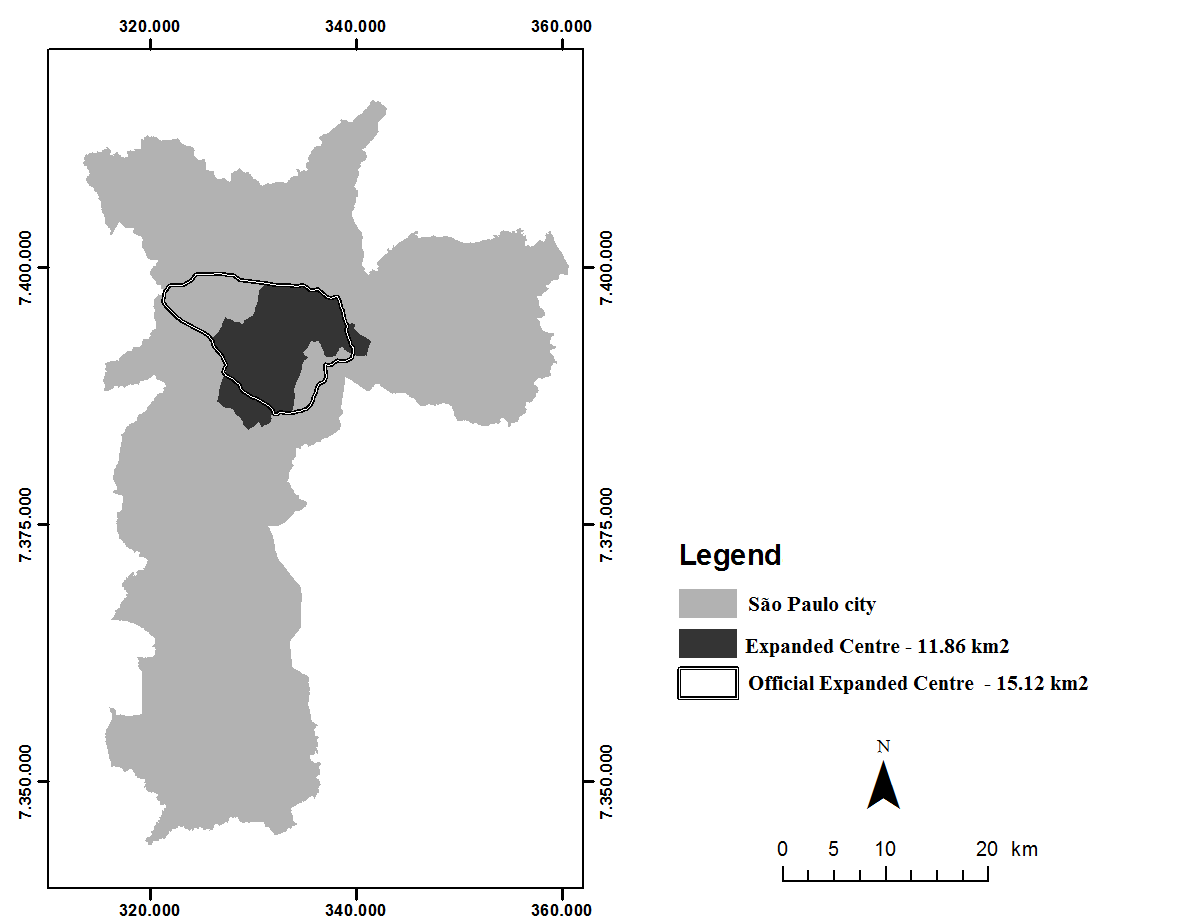


Figure S1. Geographic differences between the municipal official boundaries of the expanded central area^a^ and the area representing the expanded centre in the study.

^a^ Districts considered to be in the expanded central area: Bela Vista, Bom Retiro, Brás, Cambuci, Consolação, Liberdade, Pari, República, Santa Cecília, Sé, Água Rasa, Belém, Mooca, Jardim Paulista, Pinheiros, Campo Belo, Itaim Bibi, Moema, Saúde e Vila Mariana.


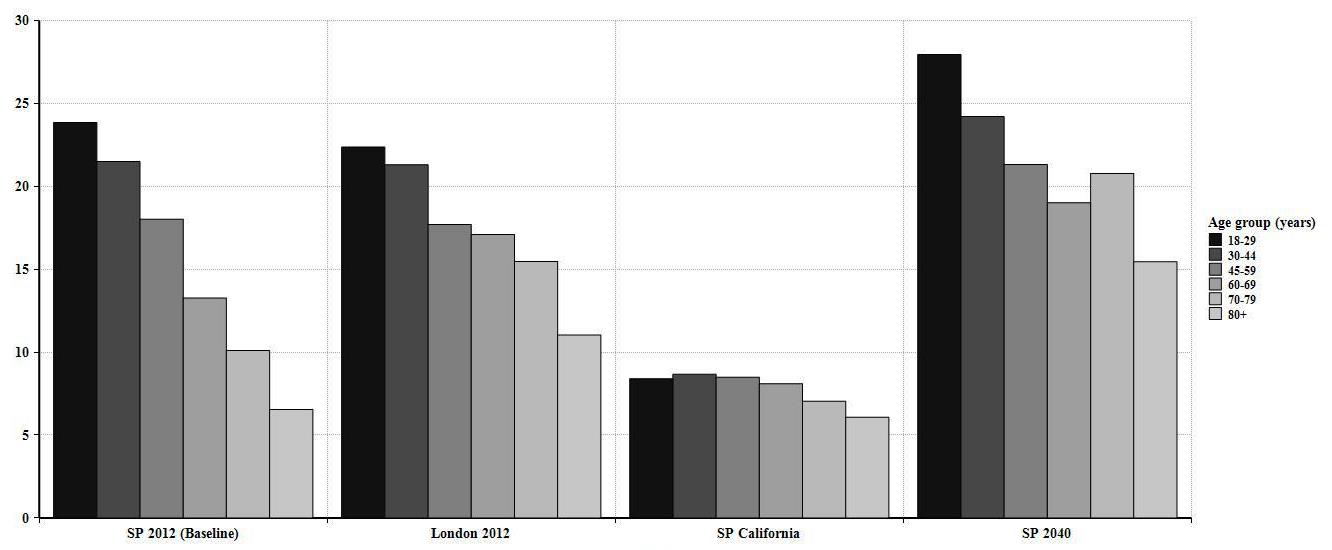


**Average minutes per day**

Figure S2. Average walking minutes per day by age groups and scenario.

.


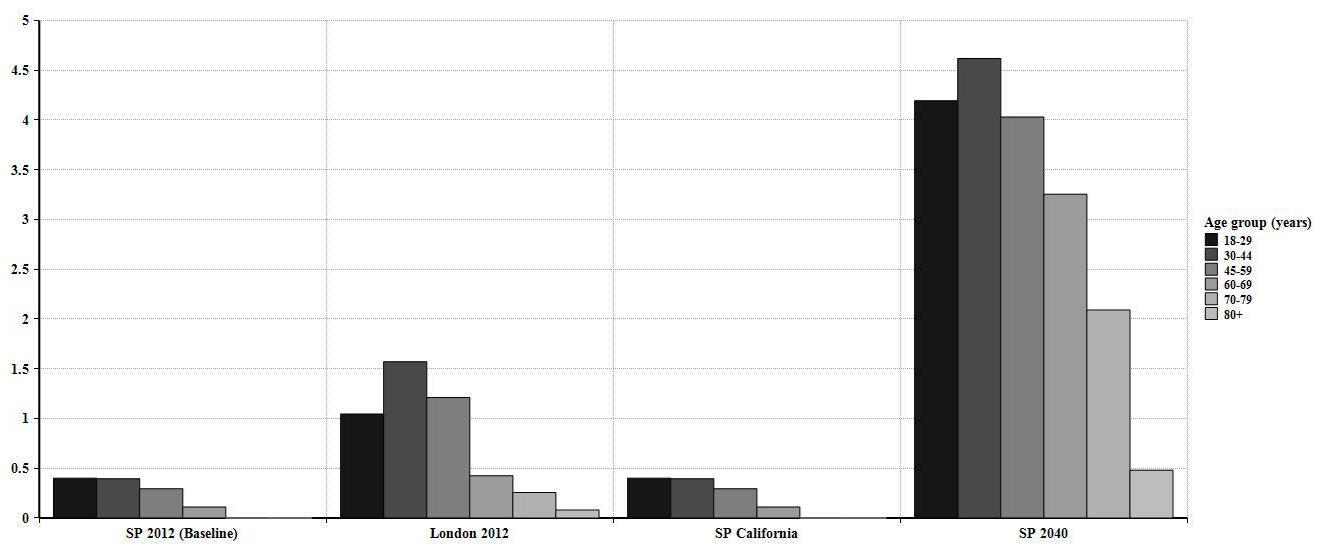


**Average minutes per day**

Figure S3. Average cycling minutes per day by age groups and scenario.

*Estimations of Carbon Dioxide equivalent greenhouse gases vehicle emissions (CO_2eq_)*

We estimated the carbon dioxide (CO_2_) equivalent greenhouse gases vehicle emissions (CO_2eq_) for São Paulo city by multiplying the vehicle and fuel type fraction of São Paulo city fleet to the CO_2eq_ by mode of São Paulo Metropolitan Area (Companhia Ambiental do Estado de São Paulo, 2013). The report from which these data was obtained (Companhia Ambiental do Estado de São Paulo, 2013) used the following gases and weights to estimate CO_2eq_: CO2 (CO_2eq_ = 1); CH_4_ (CO_2eq_ = 21); N_2_O (CO_2eq_ = 310), based on the 2006 IPCC Guidelines for National Greenhouse Gas Inventories (Intergovernmental Panel on Climate Change (IPCC), 2006). Changes in CO_2eq_ from baseline were estimated based on changes in travel time by vehicle mode for each scenario. We did not take into account emissions of black carbon, a very short lived climate pollutant, in the CO_2eq_ estimation.

Had São Paulo adopted the travel pattern of SP California, 11.4 million tonnes of CO_2eq_ emissions would be added to the current 7 million tonnes of CO_2eq_ emissions in the city (163% increase). Increases in CO_2eq_ emissions were also observed for SP EC and SP London 2012 scenarios (1.3 million tonnes and 2.1 million tonnes or 19% and 30% increases, respectively). The only scenario with reductions in CO_2eq_ emissions was SP 2040 (-3.6 million tonnes or 51% reduction).

*Sensitivity Analysis*

The impact of changes to physical activity and air pollution exposures modelled directly on all-cause mortality rather than modelling disease specific mortality is presented in Table S4. For any dose-response or RR used, DALY changes from physical activity were consistently larger when modelled directly on all-cause mortality. For instance, in the SP California scenario, DALY losses from physical activity at least doubled, being nearly five times higher when using the RR recommended for HEAT (Kahlmeier et al., 2011) (34.3k versus 158.9k). As for air pollution, modelling impact on all-cause mortality also produced much higher estimates for all scenarios, around four to five-fold higher than the disease-specific estimates. Nonetheless, overall net health impact of any scenario was always in the same direction (Table S4).

Sensitivity of the model to key parameters was presented with tornado plots for each scenario (Figures S4 to S7), illustrating health impacts from replacing the key parameter of interest with the age-gender specific 97.5^th^ and 2.5^th^ percentile of the parameter distribution. The model was most sensitive to assumptions on parameters related to physical activity, particularly the shape of the dose-response function and the values assumed for walking METs (Table S5). Other parameters most influential across scenarios were the fractions of transport PM2.5 assumed for different modes, the fraction of PM2.5 due to road transport, injury YLD uncertainty, and the relative risk for cardiovascular diseases (Table S5). The remaining parameters examined played a minor role. Irrespective of the parameter tested, the direction of net health impacts was always maintained for any scenario (Figures S4 to S7).

| Table S4. DALY comparison if calculating physical activity and air pollution impact from all-cause mortality according to different exposure-response functions (ERF). | | | | |
| --- | --- | --- | --- | --- |
|  | SP EC | SP London 2012 | SP California | SP 2040 |
| Air pollution disease-specific (same as in Figure 2) | -8665 (-11962 to -5732) | -8636 (-12402 to -5481) | -6692 (-13519 to -402) | -12234 (-16344 to -8471) |
| Air pollution ERF from Héroux et al. (2015) | -33911 (-53164 to -19855) | -23400 (-40492 to -11213) | -31146 (-63519 to -6451) | -47620 (-73273 to -28353) |
| Physical activity disease-specific (same as in Figure 2) | -3237 (-6170 to -1720) | -5608 (-13341 to -752) | 34291 (24143 to 45907) | -34363 (-58368 to -19762) |
| Physical activity ERF from Woodcock et al. (2011) | -14155 (-22514 to -8758) | -8320 (-29565 to 8552) | 136292 (104619 to 171497) | -108630 (-161673 to -70843) |
| Physical activity ERF from Wen et al. (2011) | -14132 (-16032 to -12291) | -23271 (-27818 to -18834) | 71712 (62827 to 80736) | -109245 (-121951 to -96987) |
| Physical activity walking alone ERF from HEAT (2011) | -16199 (-35010 to -1486) | -6267 (-33646 to 18362) | 158887 (12719 to 363292) | -124060 (-260170 to -11705) |
| Physical activity walking alone ERF from Woodcock et al. (2011) | -8483 (-17261 to -2877) | -5600 (-22318 to 4289) | 78548 (27355 to 135524) | -65005 (-124876 to -22708) |

| Table S5. Parameters influencing uncertainty of model results for each scenario. | | |  |  |
| --- | --- | --- | --- | --- |
|  | SP EC | SP London 2012 | SP California | SP 2040 |
| 1st | Walking MET values | Shape of PA dose-response function | Walking MET values | Shape of PA dose-response function |
| 2nd | Shape of PA dose-response function | Fraction of PM2.5 from bus | Injury YLD uncertainty | Walking MET values |
| 3rd | Injury YLD uncertainty | Fraction of PM2.5 due to road transport | RR for cardiovascular diseases | RR for cardiovascular diseases |
| 4th | Fraction of PM2.5 from bus | Safety-in-numbers uncertainty | Lifelong injury % uncertainty | Injury YLD uncertainty |
| 5th | Lifelong injury % uncertainty | RR for cardiovascular diseases | Safety-in-numbers uncertainty | Safety-in-numbers uncertainty |
| 6th | Safety-in-numbers uncertainty | Fraction of PM2.5 from cars | Fraction of PM2.5 from cars | Cycling MET values |
| 7th | Subway pollution harm | Injury YLD uncertainty | Shape of PA dose-response function | Lifelong injury % uncertainty |
| 8th | Fraction of PM2.5 due to road transport | Cycling MET values | RR for depression | RR for type 2 Diabetes |
| 9th | Subway concentrations of PM2.5 | Fraction of PM2.5 from trucks | RR for type 2 Diabetes | Injury underreporting |
| 10th | RR for cardiovascular diseases | Fraction of PM2.5 from motorcycles | Injury underreporting | Fraction of PM2.5 due to road transport |
| In gray: parameters related to air pollution. In green: parameters related to physical activity. In red: parameters related to road injury. | | | | |

| Table S6. Age and gender distribution of the adult population of São Paulo in 2012 and projections for São Paulo in 2040 (# (% Total)). | | | | | | | |
| --- | --- | --- | --- | --- | --- | --- | --- |
|  | 2012 | | |  | 2040 | | |
|  | Men | Women | Total |  | Men | Women | Total |
| 18-29 | 1050207 (12%) | 1127303 (12.9%) | 2177510 (24.8%) |  | 919453 (9.1%) | 872750 (8.6%) | 1792203 (17.7%) |
| 30-44 | 1258238 (14.4%) | 1419940 (16.2%) | 2678178 (30.6%) |  | 1456845 (14.4%) | 1410357 (14%) | 2867202 (28.4%) |
| 45-59 | 954353 (10.9%) | 1154548 (13.2%) | 2108901 (24.1%) |  | 1335783 (13.2%) | 1381643 (13.7%) | 2717426 (26.9%) |
| 60-69 | 393247 (4.5%) | 569726 (6.5%) | 962973 (11%) |  | 646254 (6.4%) | 729063 (7.2%) | 1375317 (13.6%) |
| 70-79 | 212166 (2.4%) | 317510 (3.6%) | 529676 (6%) |  | 395512 (3.9%) | 504456 (5%) | 899968 (8.9%) |
| 80+ | 114301 (1.3%) | 192224 (2.2%) | 306525 (3.5%) |  | 167419 (1.7%) | 277994 (2.8%) | 445413 (4.4%) |
| Total | 3982512 (45.4%) | 4781251 (54.6%) | 8763763 (100%) |  | 4921266 (48.7%) | 5176263 (51.3%) | 10097528 (100%) |

Table S7. Average non-travel physical activity by age and gender (marginal METh per week).

|  | Men | Women |
| --- | --- | --- |
| 18-29 | 14.9 | 7.6 |
| 30-44 | 9.0 | 3.9 |
| 45-59 | 4.0 | 3.2 |
| 60-69 | 4.6 | 3.4 |
| 70-79 | 4.1 | 2.4 |
| 80+ | 4.0 | 1.0 |

Figures S4 to S7. Tornado plots from sensitivity analysis.

Figures S4. Tornado plot for the SP Expanded Centre scenario.

Figures S5. Tornado plot for the London 2012 scenario.

Figures S6. Tornado plot for the SP California scenario.

Figures S7. Tornado plot for the SP 2040 scenario

References in the Supplementary file

Companhia Ambiental do Estado de São Paulo, 2013. Emissões veiculares no estado de São Paulo 2012. Governo do Estado de São Paulo, São Paulo, p. 108.

Intergovernmental Panel on Climate Change (IPCC), 2006. 2006 IPCC guidelines for national greenhouse gas inventories. Intergovernmental Panel on Climate Change.

Kahlmeier, S., Cavill, N., Dinsdale, H., Rutter, H., Götschi, T., Foster, C.E., 2011. Health Economic Assessment Tools (HEAT) for Walking and Cycling. Methodology and User Guide. Copenhagen: World Health Organization Regional Office for Europe.
